# Trauma is danger

**DOI:** 10.1186/1479-5876-9-92

**Published:** 2011-06-15

**Authors:** Paul F Hwang, Nancy Porterfield, Dylan Pannell, Thomas A Davis, Eric A Elster

**Affiliations:** 1 Regenerative Medicine Department, Naval Medical Research Center, Silver Spring, MD USA; 2 Department of Surgery, General Surgery Service, Walter Reed National Military Medical Center, Bethesda, MD USA; 3 Department of Surgery, Uniformed Services University of the Health Sciences, Bethesda, MD USA; 4 Canadian Forces Health Services, Department of National Defense. Ottawa, ON Canada; 5 Department of Surgery, University of Toronto, ON, Canada

## Abstract

**Background:**

Trauma is one of the leading causes of death in young adult patients. Many pre-clinical and clinical studies attempt to investigate the immunological pathways involved, however the true mediators remain to be elucidated. Herein, we attempt to describe the immunologic response to systemic trauma in the context of the Danger model.

**Data Sources:**

A literature search using PubMed was used to identify pertinent articles describing the Danger model in relation to trauma.

**Conclusions:**

Our knowledge of Danger signals in relation to traumatic injury is still limited. Danger/alarmin signals are the most proximal molecules in the immune response that have many possibilities for effector function in the innate and acquired immune systems. Having a full understanding of these molecules and their pathways would give us the ability to intervene at such an early stage and may prove to be more effective in blunting the post-injury inflammatory response unlike previously failed cytokine experiments.

## Introduction

The immune system has two effector arms, innate and adaptive, which mediate the response to pathogens and injury. The innate system is a non-specific response while the adaptive system is pathogen and antigen specific. This system has evolved to respond appropriately to pathogen or injury, but may be maladaptive in the setting of overwhelming injury as seen in complex traumatic war wounds or multisystem civilian trauma. In the setting of severe traumatic injury, the immune system is overwhelmed by the massive release of endogenous signals from injured tissue. Once systemically activated, the immune system reacts against the host, potentiating tissue damage and leading to organ failure [1]. In this situation, the immunologic response to injury, not the actual injury itself, leads to undue morbidity, and in some cases mortality.

While immune mediated responses have classically been thought to center on self and non-self interactions and thereby neglect most traumatic injuries, the Danger model abandons this classical concept [2]. The Danger model theorizes that the immune system's primary driving force is the need to detect and protect against danger and does not discriminate between self and non-self [2]. This concept states that the mechanism by which a cell dies governs whether the immune response is initiated. Therefore, tissue damage or an injury or endogenous signals of cell distress can trigger both an innate and adaptive response only if it causes danger, a non-controlled and abnormal cell death process unlike apoptosis. In the absence of danger, host tissues remain healthy or undergo apoptotic death and are scavenged, and no immune response occurs. In contrast, when an infectious or sterile insult causes cell damage, lysis or apoptosis with release of intracellular contents an immune response is initiated" [3]. Thus, the immune system is governed from within, responding to *endogenous *signals that originate from stressed or injured cells.

**Figure 1:**
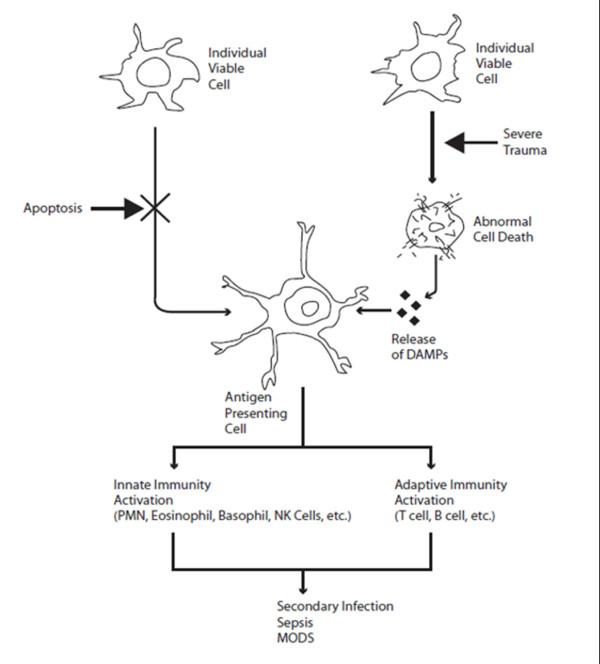
**Immunologic Response to Severe Trauma**.

Severe multi-system trauma can result in the systemic activation of the innate immune system [4]. This may result in a detrimental self-aggressive immunologic response with subsequent secondary infection, sepsis and multiple organ dysfunction (Figure 1). Various immune cell-derived mediators are produced and released during trauma, including complement factors, coagulation system factors, acute phase proteins, and neuroendocrine mediators, which have been shown to play a major role in systemic inflammation [1]. These Danger signals can activate innate immune responses after trauma [5] and also act as the initiator of further downstream effector responses through their liberation after traumatic injury and hemorrhagic shock. In this review, we attempt to describe the immunologic response to systemic trauma in the context of the Danger model with a review of the key mediators in support of this paradigm. The understanding of this response may have broad implications in the management of the severely injured patient.

## The Danger Model

Traditional theories of immune regulation stems from the work of Sir Frank Macfarlane Burnet [6,7]. Burnet postulated that immune cells have the ability to distinguish between self and non-self antigens to allow for activation and clonal selection of the adaptive immune system [8,9]. However, it was recognized that the innate immune system played a crucial role in contributing to adaptive immune response activation through antigen presenting cells and its regulation of co-stimulatory molecules [3,10,11]. Janeway expanded the classical version of the self/non-self model through his discovery of evolutionarily primitive receptors called pattern recognition receptors (PRRs) that are able to recognize and bind to conserved microbial constituents called pathogen associated molecular patterns (PAMPs) [12-14]. These PAMPs allow for differentiation of infectious antigens from noninfectious ones based on the antigen's association with infection [13]. However, it was recognized that this concept of response to primarily exogenous stimuli such as foreign antigen or bacteria was inadequate to describe other situations such as tumors and autoimmunity, and the focus of this discourse, trauma [3,13]. In an attempt to address this conceptual deficit, a modification of the self/non-self paradigm, the Danger Theory, was introduced which clarifies the immune response in the setting of traumatic injury.

The Danger Theory, proposed by Matzinger in 1994, suggests that the function of immune system is to prevent and recognize attack from harm in the context of "Danger signals" [2]. Danger theorists believe the mechanism by which a cell dies governs whether an immune response is initiated and that the immune system does not respond to non-self but rather from Danger signals from injured/dying cells [2,3]. Matzinger has argued that the immune system is governed from within through endogenous signals, later defined as alarmins, originating from cells being stressed, signifying damage [3]. Thus, inflammation in terms of the danger therapy can be considered the result of immune activation from both exogenous and endogenous danger/alarm signals. Seong and Matzinger later expanded this idea by proposing that both PAMPs and "alarmins" have similar conserved hydrophobic portions on their respective molecules, thus able to engage the same pattern recognition receptors to elicit comparable noninfectious inflammatory responses [15]. Due to their similarities, "alarmins" (which respond to endogenous signals) and PAMPs (which respond to exogenous signals) are classified as danger associated molecular patterns (DAMPs) to signify the close relationship between trauma and pathogen evoked inflammatory responses [16].

The immune response to microbial infection has a striking resemblance to the one seen in trauma. In fact, the profile of cytokine and chemokine production has been shown to be similar in the inflammatory response between sterile injury and bacterial infection [17,18]. These pathways are shared in that many endogenous Danger signals released during infection and sterile injury such as high mobility box group 1 (HMGB1), heat shock proteins (Hsp), and hyaluronan have been implicated to elicit an intrinsic inflammatory immune response through similar pattern recognition receptors [19,20]. Understanding the different Danger signals involved in sterile injury along with their mechanism may lead to possible areas of intervention and manipulation of immune responses as future therapeutic modalities.

## Singnals & Mechanism

The release of endogenous intracellular and extracellular molecules specifically generated upon tissue injury signals the threat of either infection or injury [21]. Potent immune cell activation can be mediated by so called damage associated molecular patterns via pattern recognition receptors (PPRs) such as Toll-like receptors (TLRs). TLRs represent a key molecular link between tissue injury, infection, and inflammation. Moreover, DAMPs have also been implicated in diseases where excessive inflammation plays a key role in pathogenesis [21]. Rock and Kono [13] outlined four fundamental biological outcomes that a DAMP must fulfill.

1) The purified molecule should cause an inflammatory response when injected into a living organism.

2) The purified molecule should also be active at normal physiological concentrations.

3) Microbial contamination should be ruled out as the source of inflammatory response. This is especially important in DAMPs that work through Toll-like receptors (TLRs), since these are known to sense microbial products.

4) Eliminating or neutralizing the molecule from dead cells should reduce the inflammatory response. This last criterion is most likely the hardest since it is likely that multiple extracellular matrix and/or endogenous intracellular molecules are released by activated or necrotic cells upon injury or degraded following tissue damage.

There are many molecules that have been identified as danger associated molecular patterns in the literature to include but not limited to HMGB1, Hsp, uric acid (UA), galectins, thioredoxin, adenosine, etc. [13], but here we will only examine four of the most common and well-defined, as well as their interactions with the members of the TLR family of receptors.

### High Mobility Group Box 1

**Figure 2:**
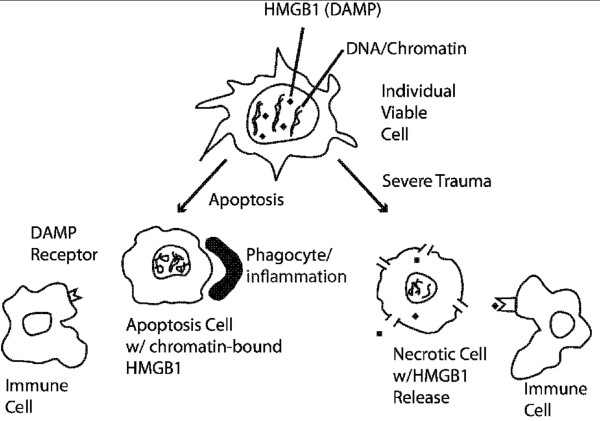
**Mechanism of HMGB1 (DAMP) Release and Immune Cell Activation**.

HMGB1 is a nuclear non-histone chromosomal protein that binds to DNA causing it to bend [7]. While intracellular HMGB1 stabilizes nucleosome formation and facilitates transcription, its extracellular release in response to inflammatory stimuli and/or tissue damage and its role as a known danger/alarmin signal-activator of innate immunity, is of interest. When released extracellularly HMGB1 functions as a potent cytokine-like factor driving the initiation and perpetuation of other proinflammatory mediators, inducing cell-mediated inflammatory (Th1 type) responses, and serves as well as a chemo-attractant for immature dendritic cells which process and present antigen [22]. In contrast to necrotic cells, cells undergoing apoptosis retain HMGB1 irreversibly bound to their chromatin and do not support inflammation [23]. When a few apoptotic cells are cleared by macrophages, HMGB1 is released but does not seem to stimulate an immune response [24]. However, when a large number of apoptotic cells are cleared in this pattern, there is a large level of HMGB1 passively released in addition to that actively secreted from a variety of HMGB1 secreting cell types following this inflammatory stimulus [16]. In contrast, when cell membrane integrity is lost, as happens with tissue injury or cell necrosis, HMGB1 is released into extracellular space and signals danger to the surrounding cells (Figure 2). Active secretion acts as a pro-inflammatory cytokine during an immunological challenge, orchestrating a defensive inflammatory response to ischemia, burn, infection or sepsis and initiate tissue regeneration [25]. In vitro, HMGB1 released from necrotic cells stimulated production of TNF-α, a pro-inflammatory cytokine [13]. Tsung et al. [26] demonstrated that injection of HMGB1 increased tissue damage after hepatic ischemia reperfusion injuries. It has also been demonstrated that administration of HMGB1 antibodies reduced inflammation and provided some protection from injury in both ischemic reperfusion [26] and thermal burns [27]. There is some concern that the inflammatory reaction stimulated directly by HMGB1 is inconsistently reproduced [16]. Some studies have shown that HMGB1 injected into the heart after an infarction can promote regeneration and recovery of the cardiac performance [28]. Although this goes against the fourth tenet defining a DAMP, this is simply the end result which involves a complex pathway involving RAGE receptor interrogation and c-kit+ cardiac cells that play a key role in cell proliferation and differentiation *in vivo *[28]. Preventing, blocking and/or neutralizing HMGB1 release by injured cells is a compelling active area of research focus and could potentially become a therapeutic avenue for intervention.

While HMGB1 can influence the initiation of both innate and adaptive immune responses, the mechanism by which HMGB1 functions as a DAMP is poorly defined. Huang et al. [27] demonstrate that the antibody against the receptor for advanced glycation end products (RAGE) inhibits the ability of HMGB1 to promote inflammation. This implicates RAGE as a receptor that binds HMGB1 and signals inflammation. Similarly, S100A12 and S100B, a subset of calcium binding proteins also with potential to be a DAMP, interact with RAGE to induce a specific inflammatory pattern with increased vascularity in endothelial cells and a prothrombotic effect, although these effector functions presence after HMGB1 activtion is yet to be determined [16]. The same group has demonstrated that administering HMGB1 blocking agents, such as ethyl pyruvate or anti-RAGE, can significantly reduce serum levels of HMGB1 and restore the expression levels of IL-2 and IL-2a, which mediate expansion of T-cells, key players in the adaptive response. In this study the expression levels of CD152 and Foxp3 were elevated on splenic regulatory T cells, but expression levels of both markers were reduced in groups that were administered ethyl pyruvate or anti-RAGE [27]. With respect to the early inflammatory response Tsung et al. [26] demonstrated that tissue damage caused by hepatic ischemia was decreased when mice were treated with neutralizing antibodies against HMGB1. Endogeneous tissue damage was worsened when additional exogenous HMGB1, in the form of recombinant HMGB1, was administered to mice after hepatic ischemic injuries [26]. This demonstrates that HMGB1 acts as an early danger/alarmin signal and mediator of tissue injury and trauma in liver ischemia [26]. This could be extrapolated out to surgical trauma but this has yet to be demonstrated in pre-clinical models. HMGB1 has already been proven to be a successful therapeutic target in experimental models of infectious and inflammatory disorders including sepsis, cancer and theumatoid arthritis [25], we are getting closer to isolating this molecule as a target for therapeutic manipulation in trauma.

### Heat Shock Proteins

Hsp are intracellular cytoprotective chaperone proteins that play key roles in intracellular trafficking, protein folding and maintenance of protein integrity during normal and stress-induced environmental conditions [29]. Hsp's are released from a variety of cell types, present on cell surfaces and found in the serum [30]. The upregulation and extracellular release of heat shock proteins acts as a Danger signal in response to stresses involving cell necrosis from innate immune reactions encompassing bacterial infections/antigen and/or the clearance of neoplastic-transformed cells [29]. When released in response to stress Hsp's provide protection against apoptosis via both upstream and downstream pathways [31]. This potentially allows the cell to continue an inflammatory response. Prohaska et al [32]. reported that stressed-induced extracelleular Hsp70 induces pro-inflammatory responses in human monocytes [32]. Hsp70 released into the extracellular milieu specifically binds to Toll-like receptors (TLR2 and TRL4) on antigen-presenting cells (APC) through a CD14-dependent pathway and exerts immunoregulatory effects, including the upregulation of adhesion molecules, co-stimulatory molecule expression, and cytokine and chemokine secretion [33]. Interestingly, dendritic cells are capable of distinguishing the stressed apoptotic cells versus the non-stressed cells based on the presence of heat shock proteins on the plasma membrane [30]. Although further studies are needed to determine the exact mechanism and effector functions caused by the activation of dendritic cells by Hsp, Campisi et al. [34] reported that enhanced production of nitric oxide, TNF-α, IL-1β, and IL-6 in rat macrophages and splenocytes response with Hsp72 stimulation. Moreover, nitric oxide and cytokine responses were further augmented when cells were exposed to the combination of Hsp72 plus LPS. This robust response required five times less Hsp72 than LPS to produce a nearly equivalent response and evidence has been reported to show this was not due to endotoxin contamination [34]. Hsp's could be considered potential targets to prevent tissue injury caused by trauma-induced cellular stress through this cytokine activation pathway.

### Monosodium Urate

UA is an important Danger signal, whose effects are mediated by its extracellular release from activated or necrotic cells. UA has been shown to mediate both innate and adaptive immune regulatory responses. The active form of the molecule, monosodium urate crystals, can trigger inflammation and has been to act as an adjuvant in promoting dendritic cell maturation and activating dendritic cell mediated immune responses [16,35]. In vitro, the uptake of monosodium urate crystals by monocytes involves interactions with Toll like receptors, specifically TLR2 and TLR4 [36]. Furthermore, the presence of intracellular monosodium urate crystals has been shown to activate the innate immune system thru a range of receptors, specifically of the TLR family, and proteins that detect pathogens along with damaged or dying cells through pattern recognition motifs [36]. This in turn leads to the formation of an inflammasome complexes that responds to IL-1 to yield mature IL-1β to be secreted [36]. The inflammatory affects of monosodium urate crystals have been shown to be blocked by IL-1 inhibition, leading to a rapid and dramatic effect on the signs and symptoms of inflammation [37]. Thus, monosodium urate exemplifies the definition of a DAMP thru its activation of the innate system.

### RNA/DNA

CpG rich regions of RNA and DNA have been shown to bind to TLRs and stimulate cytokine production, and therefore function as Danger signals. Ishii et al. [38] demonstrated that double stranded DNA enhanced antigen presenting cell function *in vitro *and improved primary cellular and humoral immune response in vivo. This response was dependent on the length and concentration of double stranded DNA but were independent of sequence [38]. As mentioned above one of the important criteria in identifying a danger associated molecular pattern is ensuring the purified danger associated molecular pattern is free of endotoxins. When the double stranded DNA is reduced to single stranded the ability to induce antigen presenting cell maturation is lost [38]. Antigen presenting cell maturation was also induced by CpG-containing bacterial DNA in both single and double stranded DNA formats [38]. When the single-stranded bacterial DNA is methylated at the CpG motifs it no longer capable of stimulating antigen presenting cell maturation [38].

Mitochondria and their related moieties are an important set of molecules that may play a role as DAMPs during sterile inflammation and injury. Because they are thought to have a genetic makeup that is bacterial in origin, in theory, injury to cellular structures allowing for the release of mitochondrial contents into the bloodstream that would normally stay hidden [39]. Just like PAMPs, these damage associated mitochondrial patterns become recognized by PRRs and start the cascade of inflammatory and immune mediators with eventual SIRS reaction [40]. This pathway showing release of mitochondrial contents during injury and leading to neutrophil migration and degranulation through a TLR mediated mechanism with subsequent organ injury, has been show before [40]. Even surgical trauma from femur fractures showed release of mitochondrial damage associated molecular patterns that have the ability to activate polymorphoneuclear cells in rats, specifically in the lung [41]. Although the lung injury induced was not as severe, this does support mitochondrial damage associated molecular patterns serving as a priming stumulus that when hit with a second stimulus, can lead to further injury to the end organ with increasing inflammatory and immunological cascade effects and increased severity of injury [39].

### Toll-like Receptors

The common molecular pathway between sterile injury, infection and the inflammatory response is thought to be mediated by stimulation of Toll-Like Receptors (TLRs) [21]. This family of receptors displays homology to the Drosophila melanogaster *Toll *gene product [42] which is involved in embryogenesis and immunity. By study of mutations in murine homologues it was shown that TLR4 is the receptor responsible for the recognition and inflammatory response to LPS [43]. Subsequent studies have demonstrated there are approximately12 human TLR homologues and nine murine homologues [21,44]. These receptors recognize both exogenous and endogenous Danger molecules as ligands that subsequently lead to one of two distinct signaling cascades that culminate in an activated host inflammatory response. TLRs recognize a wide variety of exogenous ligands (PAMPs) through leucine-rich repeats located in their extracellular domains [45]. Three TLR ligand-receptor interactions have been elucidited: TLR3/dsRNA [46], TLR1-TLR2 heterodimers bound to the Pam_3_CSK_4 _lipopeptide [47], and TLR4/LPS via the co-receptor MD-2 [48]. These and other experiments have shown that TLRs recognize PAMPs via diverse mechanisms involving homodimerization, heterodimerization, direct ligand-receptor interactions, accessory molecules and co-receptors. Further, these multiple complex interactions help to account for the ability of TLRs to recognize such a wide array of Danger molecules.

Endogenous DAMP-TLR interactions have been reported *in vitro*, by utilization of immunoprecipitation assays, in cell culture experiments, and *in vivo *using murine models with targeted mutations. For example, the heat shock proteins Hsp60 and Hsp70 have been shown to interact directly with TLR-2 and TLR-4 causing activation of mononuclear cells [49], and HMGB1 requires the co-receptor MD-2 to activate TLR-2 and TLR-4 [50]. As mentioned previously, monosodium urate uptake by monocytes has also been shown to be mediated by interaction with TLR-2 and TLR-4, whereas dsDNA containing immune complexes cause dendritic cell maturation through activation of TLR-9. Future studies using fluorescence resonance energy transfer microscopy and GFP fragment reconstitution to demonstrate molecular proximity have been proposed [51] and may provide further *in vivo *evidence to identify interactions between endogenous DAMPs and TLR receptors.

When bound by their ligands or ligand complexes, TLRs are known to activate two distinct signalling pathways involved in inflammation. The first uses the signaling adaptor molecule myeloid differentiation factor 88 (MyD88) and is activated by all TLRs with the exception of TLR3. This signaling cascade is propagated by various IL-1 receptor associated kinases and mitogen activated protein kinases and results in NF-κβ activation which in turn acts as a direct or indirect (via inflammatory cells) transcriptional activator of pro-inflammatory cytokine and chemokine (IL-1α/β, IL-6, IL-8, MIP-1α/β, TNF-α,) gene expression [16]. This common final pathway may be the link between sterile tissue injury and infections thru pathogens, thus allowing us to further understand the true mechanism behind trauma evoked immunological response. The second pathway, activated by ligand binding of TLR3 and TLR4 is MyD88 independent, and culminates in the transcriptional activation of interferon (IFN) [21]. Induction of IFN expression is an additional pathway that allows TLRs to synthesize multiple mediators of inflammatory and immune responses allowing for specific cellular responses [21].

Signaling mediated by different TLR pathways has been demonstrated to lead to different functional responses. This suggests that TLR signaling is capable of differential immune responses given varying stimuli whether from endogenous or exogenous DAMPs [21]. For example, recent studies have shown that HSP60 and LPS cause differential activation of APC function [52], and that HMGB1 activation of neutrophils causes up-regulation Bcl-xl and monoamine oxidase B, which is not seen in LPS stimulation [53]. Further, microarray experiments have demonstrated differential inflammatory gene activation in MH-S cells when stimulated with either the DAMP hyaluronic acid and LPS [54].

These data support a model where immune stimulation by exogenous (PAMP's) and endogenous ("alarmins") DAMPs activate different end pathways [16]. Therefore, different targets for intervention between the inflammatory response to sterile traumatic or infectious insult may exist and targeting one alone may help decrease pathological inflammatory response to injury while keeping host immune response to infection intact. Further elucidation of the poorly described intracellular signaling pathways downstream of TLR activation by DAMPs may provide insight into key strategies for modulating maladaptive TLR activation in the injured patient, while maintaining immunocompetence.

## Conclusions & Future Directions

Despite the large amount of research dedicated to multifunctional danger/alarm signals, much still remains to be elucidated prior to any discovery of pathways for therapeutic and immunomodulatory action. In this review, we have described some of the pathways whereby Danger molecules lead to an activation of the innate which causes local inflammation and recruit cells of the innate immune system and subsequent release of pro-inflammatory cytokines. It is important to note that activation of this pathway in turn may results in generation of a systemic inflammatory response syndrome (SIRS). In the traumatically injured critically ill patient this occurs as tissue injury leads to cell necrosis and release of Danger signals. These DAMPs are thought to activate TLRs triggering the innate immune response to release cytokines and other pro-inflammatory mediators (such as IFN) causing the clinical syndrome of SIRS. Indeed, plasma levels of HMGB1 after severe injury have been shown to correlate with development of SIRS, and early elevation of HMGB1 is associated with increased mortality [55].

If traumatic SIRS is not attenuated by the compensatory anti-inflammatory response syndrome (CARS) a deleterious pro-inflammatory cascade may ensue, potentially resulting in MODS and death [56]. Therefore, it is evident that further investigation of the exact mechanism and role that Danger molecules play in this process is central for preventing morbidity and mortality associated with traumatic injury. Identifying the pathways involved in the inflammatory response to injury would enable clinicians to differentiate sterile SIRS from sepsis and allow for a tailored approach to treatment. Further, identifying which patients are most likely to develop severe SIRS after injury may allow for early intervention.

Our current knowledge of Danger signals is incomplete and this knowledge gap continues to expand as new ones emerge. Others that have been added in the literature thus far include galectins, thymosins, nucleolins, annexins, and thioredoxin [16,57-61], all whose kinetics, mechanisms, and associations with severe trauma are still unknown.

Future clinical studies need to be completed to evaluate Danger signals and their associations with outcomes in trauma. Jastrow et al. [62] provided insight into the predictive value of cytokine production as an index for developing future outcomes of multiple organ dysfunction or failure. As danger/alarmin signals are released in the acute setting after massive injury, they are the earliest markers of inflammation and may serve to predict outcomes earlier than other biomarkers. Previous clinical studies have evaluated the correlation between HMGB1 or Hsp and with outcomes such as survival and acute lung injury, with intriguing results [63,64]. Future research may wish to focus on the earlier detection of HMBG1, Hsp, and other Danger signals along with correlation to various other outcomes to include multiple organ failure and survival. This would be important to ascertain the predictive value of detecting Danger signals versus other previously evaluated biomarkers of interest.

Finally, clinical trials will be necessary to evaluate for the possible use of immunomodulation of Danger signals. Previous pre-clinical experiments in sepsis and trauma have focused on downstream cytokines in order to emulate human response [65-68]. These models demonstrated a decreased inflammatory response when TNF-α and IL-1 inhibitors were administered following an endotoxin or gram negative bacteria challenge [66]. Although promising, these results did not translate into changes in practice as they failed to demonstrate a decrease in the mortality outcome in Phase II and III studies [69,70]. One can argue that these studies focused on downstream cytokines and to really have some effect, one needs to look to more proximal signaling mechanisms to have a therapeutic effect. Even Recombinent activated protein C (Xigris^®^), although approved by the FDA in 2001 for patients with severe sepsis, some subsequent studies showed a lack of efficacy and increased incidence of bleeding in general clinical use [71]. Danger/alarmin signals are the most proximal molecules in the immune response that have many possibilities for effector function in the innate and acquired immune systems. Having a full understanding of these molecules and their pathways would give us the ability to intervene at such an early stage and may prove to be more effective in blunting the post-injury inflammatory response unlike previously failed cytokine experiments. The impact of effective strategies to limit the immune response following traumatic injury may be limitless. Nevertheless, we are not at that stage and much still remains to be elucidated before these therapeutic strategies can be effective in reality.

## Disclaimer

The views expressed in this manuscript are those of the authors and do not reflect the official policy of the U.S. Department of the Army, U.S. Department of the Navy, the U.S. Department of Defense, Canadian Forces Health Services, Canadian Department of National Defense, or the United States & Canadian Governments.

Some of the authors are U.S and Canadian military service members (or employee of the U.S. Government). This work was prepared as part of our official duties. Title 17 U.S.C. 105 provides the "Copyright protection under this title is not available for any work of the United States Government." Title 17 U.S.C. 101 defines a U.S. Government work as a work prepared by a military service member or employee of the U.S. Government as part of that person's official duties.

I/We certify that all individuals who qualify as authors have been listed; each has participated in the conception and design of this work, the analysis of data (when applicable), the writing of the document, and the approval of the submission of this version; that the document represents valid work; that if we used information derived from another source, we obtained all necessary approvals to use it and made appropriate acknowledgements in the document; and that each takes public responsibility for it.

## Competing interests

The authors declare that they have no competing interests.

## Authors' contributions

PFH, NKP, DP, TD, and EE participated in the conception and design of the manuscript, drafting of the manuscript, critical review and editing of the manuscript. All authors read and approved the final manuscript.
